# Association of glycemic control with hypertension in patients with diabetes: a population-based longitudinal study

**DOI:** 10.1186/s12872-023-03478-3

**Published:** 2023-10-10

**Authors:** Shengliang Chen, Yi Zhu, Sihui Jin, Dongbao Zhao, Jianwei Guo, Lijin Chen, Yixiang Huang

**Affiliations:** Department of Health Policy and Management, School of Public Health, Sun Yat-sen University, 74, Zhongshan 2nd Road, Guangzhou, 510030 Guangdong P. R. China

**Keywords:** Diabetes mellitus, Hypertension, Glycemic Control

## Abstract

**Background:**

Diabetes increases the risk of hypertension morbidity, but whether this association is varied with glycemic control remains unknown. We aimed to examine the association of glycemic control with hypertension among individuals with diabetes.

**Methods:**

Data was from the China Health and Retirement Longitudinal Study (CHARLS) between 2011 and 2018. Participants were categorized as having adequate glycemic control (HbA1c < 7%) and inadequate glycemic uncontrol (HbA1c ≥ 7%) by combining blood glucose tests and physician’s diagnoses in 2011. Incident hypertension was ascertained through self-reported physician diagnoses from 2011 to 2018. Cox proportional hazards regression models were used to examine the effect of glycemic control on hypertension.

**Results:**

Among 436 participants with diabetes in this study, 102 met the glycemic control standard, and 334 were insufficient glycemic control. During 7 years of follow-up, 141 individuals developed hypertension. Compared with adequate glycemic control, the hazard ratio of inadequate glycemic control on hypertension was 1.54 (95% CI, 1.07–2.21) in the multivariate model. Additionally, the influence of glycemic control on hypertension varied based on educational attainment and the presence of depressive symptoms (P for interaction < 0.05).

**Conclusions:**

Insufficient glycemic control was associated with a higher risk of hypertension among individuals with diabetes. Notably, the effect of glycemic control on hypertension was more pronounced among those with lower educational attainment and those exhibiting depressive symptoms. These findings underscore the significance of vigilant glycemic monitoring, educational background considerations, and mental health assessments in managing diabetic individuals.

**Supplementary Information:**

The online version contains supplementary material available at 10.1186/s12872-023-03478-3.

## Introduction

Diabetes mellitus, one of the most severe and common chronic diseases of the 21st century, has become a global public threat [1]. It was estimated that 10.5% of the population aged 20–79 had diabetes in 2021, rising to 12.2% in 2045 [2]. Hypertension is the most frequent comorbidity of diabetes, with over two-thirds of patients with type 2 diabetes also having hypertension [3]. The co-exist of diabetes and hypertension not only accelerates the progression of diabetes complications but also is associated with a higher risk of cardiovascular mortality. Therefore, it is of great public health significance to clarify the relationship between diabetes and hypertension.

Several studies [4–12] showed that higher fasting blood glucose was an independent risk for developing hypertension. A cross-sectional study [11] of a population of 2092 Chinese individuals aged over 65 years showed that hyperglycemia was associated with a higher prevalence of hypertension. A prospective study [12] showed that more elevated fasting blood glucose was an independent risk for hypertension among 13,201 Japanese participants. These studies mainly focused on the effect of higher blood glucose on hypertension among individuals without diabetes. However, whether this association will be affected by glycemic control in patients with diabetes remains unclear. Limited studies reported an inverse relationship between glycemic control and high blood pressure among individuals with diabetes, and no relative research was done in China [13]. Thus, the study aimed to examine the association of glycemic control with hypertension in adults with diabetes.

## Methods

### Data sources

This study used blood samples, household demographics, and health status data from the China Health and Retirement Longitudinal Study (CHARLS). CHARLS is a nationally representative population-based survey designed to research social, health, and economic issues of residents aged 45 and over [14]. CHARLS was launched in 2011, and 3 follow-up surveys have been completed since. Currently, the survey has been conducted in four waves. The first wave (W1) was conducted between 2011 and 2012, including 17,708 respondents in 10,257 households in 450 villages/urban communities in 150 counties/districts in 28 provinces. The second wave (W2) was conducted between 2013 and 2014 and included a refreshment sample of 15,770 individuals. The third wave (W3) was conducted between 2015 and 2016 and had a refreshment sample of 13,002 individuals. The fourth wave (W4) was conducted between 2018 and 2019 with a sample of 11,981 people. The details of the design and methods of CHARLS have been described extensively and can be accessed through the official website (charls.charlsdata.com).

### Study sample

**Fig. 1:**
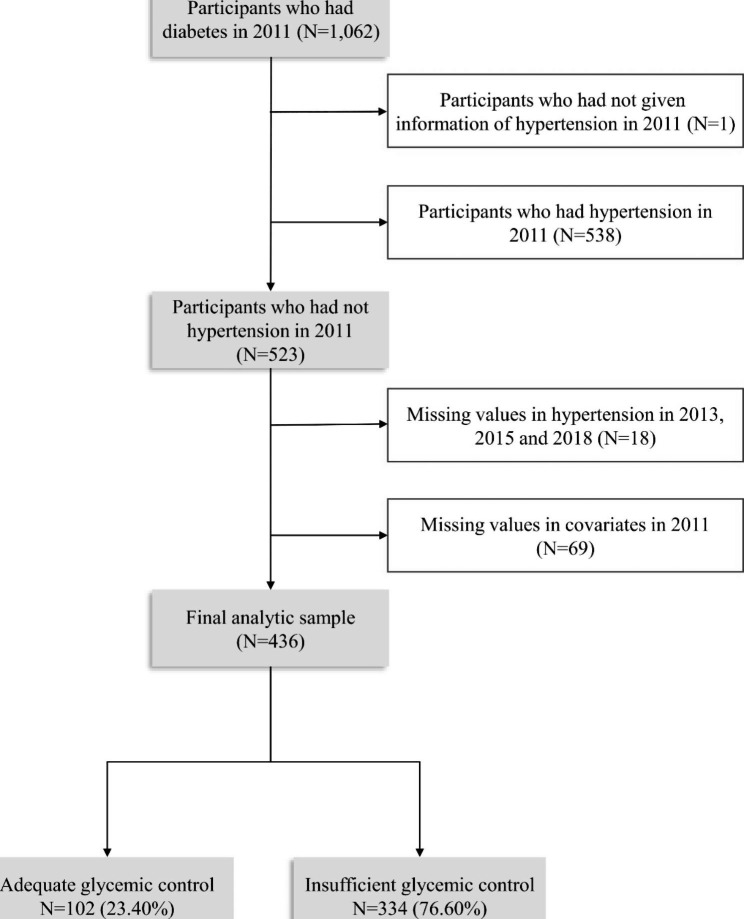
Flowchart of the study sample of Chinese middle-aged and older adults: CHARLS, 2011–2018

For this study, we used longitudinal data based on four rounds of surveys in CHARLS. This study initially selected 1062 diabetes participants with information on blood samples and aged ≥ 45 years at baseline. After excluding those lost to follow-up, without complete information on hypertension or missing data in covariates in 2011, 436 individuals were available for analysis in the present study. 436 individuals were followed up, providing complete data for all study variables. More details of the sample selection are shown in Fig. 1.

### Definitions of adequate glycemic control and insufficient glycemic control

The status of diabetes was measured by the self-reported physician’s diagnosis and blood-based bioassays and was divided into two groups, including “adequate glycemic control” and “insufficient glycemic control.“ Glycated hemoglobin A1c (HbA1c), which reflects the average blood glucose level over the preceding 3 months, is often used to monitor glycemic control [15]. Thus, we chose an HbA1c level of 7% as it represents the optimal cutoff associated with the management of diabetes in previous studies and corresponds with the target for glycemic control set by the American Diabetes Association [16]. The guideline for preventing and treating type 2 diabetes mellitus in China (2020 Edition) also recommends that the HbA1c control target for most non-pregnant adult T2DM patients is < 7%. Then, participants who admitted a history of diagnosed diabetes and had HbA1c above 7.0% in 2011 were classified as having “insufficient glycemic control.“ Participants who admitted a history of diagnosed diabetes and had HbA1c of 7.0% or less in 2011 were classified as having “adequate glycemic control.“

### Assessment of depressive symptoms

Depressive symptoms in the past week were assessed using the 10-item Center for Epidemiological Studies Depression Scale (CESD-10), a widely-used, self-reported, brief version of the original 20-item version of CESD [17]. CESD-10 contains eight negative questions and two positive questions, with answers on a four-scale metric, from rarely or none (< 1 day), to not much (1–2 days), to sometimes or half of the time (3–4 days), to most of the time (5–7 days). The four options for the negative questions were assigned values from 0 to 3, while the positive questions were assigned values from 3 to 0. The total score ranges from 0 to 30. We used universal criterion (scores ≥ 10) to identify individuals with significant depressive symptoms [18].

### Research variables

The outcome was defined as a binary variable: having hypertension or not. The question “Have you been diagnosed with hypertension by a doctor?“ demined participants’ hypertension status. The independent variable was diabetes, defined by two categorical variables: adequate glycemic control (defined as HbA1c less than 7% in 2011) and insufficient glycemic control (defined as HbA1c over 7% in 2011 and 2015). Controlled variables for this study consisted of age (categorized into age groups of 45–65, and ≥ 65years), sex (male/ female), residence (urban/ rural), marital status (married/ never married/ separated/ divorced/ widowed), drinking (yes/ no), smoking (yes/ no), education attainment (junior high school or below /junior school above), depressive symptoms (yes/no) and body mass index (BMI; underweight: BMI < 18.5 kg/m^2^; normal weight: BMI 18.5–23.9 kg/ m^2^; overweight / obesity: BMI ≥ 24.0 kg/ m^2^) [19]. All control variables were self-reported by the questionnaire.

### Statistical analysis

All the analyses were conducted by Stata V.16.0 (StataCorp, College Station, Texas, USA). Variables were compared using the Pearson chi-square test to evaluate baseline heterogeneity. We used Cox proportional hazards regression models to examine the effect of insufficient glycemic control on the incidence of hypertension. We deemed the follow-up time as the time elapsed from the date of the baseline interview to either the date of diagnosis of hypertension or the last interview in which the individual participated. We conducted six models, and the first is an unadjusted model. The second model is adjusted for age, gender, marital status, and educational attainment. The other four models are gradually adjusted for smoking, drinking, body mass index, depressive symptoms, and residence to estimate the effect of insufficient glycemic control on hypertension.

Furthermore, we conducted subgroup analyses stratified by gender, residence, education attainment, body mass index, and depressive symptoms separately. The significance level was accepted as P < 0.05 (two-sided) for all tests.

## Results

### Characteristics of participants

Table 1 shows the baseline characteristics of diabetes according to their levels of glycosylated hemoglobin A1c. Among 436 participants in this study, 102 had glycosylated hemoglobin A1c levels below 7%, representing 23.40% of the participants. Three hundred thirty-four had glycosylated hemoglobin A1c levels above 7%, representing 76.60% of the participants. Diabetes with insufficient glycemic control was more likely to be urban dwellers. Overall, baseline characteristics were well-matched between the groups.

**Table 1:** Characteristics of Chinese adults aged 45 years old and above by diabetes: CHARLS, 2011

Characteristics	N (%)	Adequate glycemic control (%)	Insufficient glycemic control (%)	P
**Total**	436 (100.00)	102 (23.40)	334 (76.60)	
**Age, years**				0.79
45–65	329 (75.46)	78 (76.47)	251 (75.15)	
> 65	107 (24.54)	24 (23.53)	83 (24.85)	
**Sex**				0.26
Male	188 (43.12)	39 (38.24)	149 (44.61)	
Female	248 (56.88)	63 (61.76)	185 (55.39)	
**Residency**				0.01
Rural	198 (45.41)	58 (56.86)	140 (41.92)	
Urban	238 (54.59)	44 (43.14)	194 (58.08)	
**Marital status**				0.93
Married	362 (83.03)	85 (83.33)	277 (82.93)	
Never married/ separated/divorced/widowed	74 (16.97)	17 (16.67)	57 (17.07)	
**Educational attainment**				
Primary school or below	274 (62.84)	73 (71.57)	201 (60.18)	0.04
Middle school and above	162 (37.16)	29 (28.43)	133 (39.82)	
**Smoking status**				0.42
Yes	147 (33.72)	31 (30.39)	116 (34.73)	
No	289 (66.28)	71 (69.61)	218 (65.27)	
**Alcohol consumption**				0.96
Yes	159 (36.47)	37 (36.27)	122 (36.53)	
No	277 (63.53)	65 (63.73)	212 (63.47)	
**BMI, kg/m** ^**2**^				0.80
Underweight (< 18.5)	26 (5.96)	7 (6.86)	19 (5.69)	
Normal (18.5–23.9)	147 (33.72)	32 (31.37)	147 (33.47)	
Overweight/Obese (≥ 24)	263 (60.32)	63 (61.76)	263 (60.32)	
**Depressive symptoms**				0.72
Yes	190 (43.58)	46 (45.10)	144 (43.11)	
No	246 (56.42)	56 (54.90)	190 (56.89)	

Table 2 shows the incidence of hypertension by diabetes in 2011–2018. In the total samples, the incidence rates of hypertension for patients with insufficient glycemic control (55.18 per 1000 person-years) were higher than those with adequate glycemic control (40.60 per 1000 person-years).

**Table 2:** Incidence of hypertension per 1000 person-years by diabetes, 2011–2018

Incidence	Adequate glycemic control	Insufficient glycemic control
Total		
Incidence cases (n)	27	114
Follow-up (person-years)	665	2066
Incidence rate of hypertension	40.60	55.18

### Risk of hypertension for diabetic patients with insufficient glycemic control

Table 3 illustrates the Cox proportional hazards regressions on the effect of glycemic control on incident hypertension. The risk of incident hypertension was significantly higher in patients with insufficient glycemic control than in those with adequate glycemic control, with a hazard ratio (HR) of 1.46 (95% CI: 1.06, 2.01). In the maximum adjustment model, incident hypertension remained higher in patients with insufficient glycemic control than those with adequate glycemic control, with a hazard ratio (HR) of 1.54 (95% CI: 1.07, 2.21).

**Table 3:** The hazard ratio of hypertension in Chinese mid-aged and older adults, by glycemic control: 2011–2018

Model	Adequate glycemic control HR (95%CI)	Insufficient glycemic control HR (95%CI)
**1. Unadjusted** ^**a**^	1.00	1.46 (1.06,2.01) ^*^
**2.Base** ^**b**^	1.00	1.53 (1.06, 2.20) ^*^
**3.Health factors** ^**c**^	1.00	1.50 (1.04, 2.16) ^*^
**4.BMI** ^**d**^	1.00	1.54 (1.07, 2.21) ^*^
**5.Depressive symptoms** ^**e**^	1.00	1.54 (1.07, 2.21) ^*^

**Notes**:

a. Unadjusted model

b. Adjusted for model 1 criteria and age, gender, marital status, residency, educational attainment

c. Adjusted for model 2 criteria and smoking and drinking history

d. Adjusted for model 3 criteria and BMI.

e. Adjusted for model 4 criteria and depressive symptoms

### Subgroup analyses

**Fig. 2:**
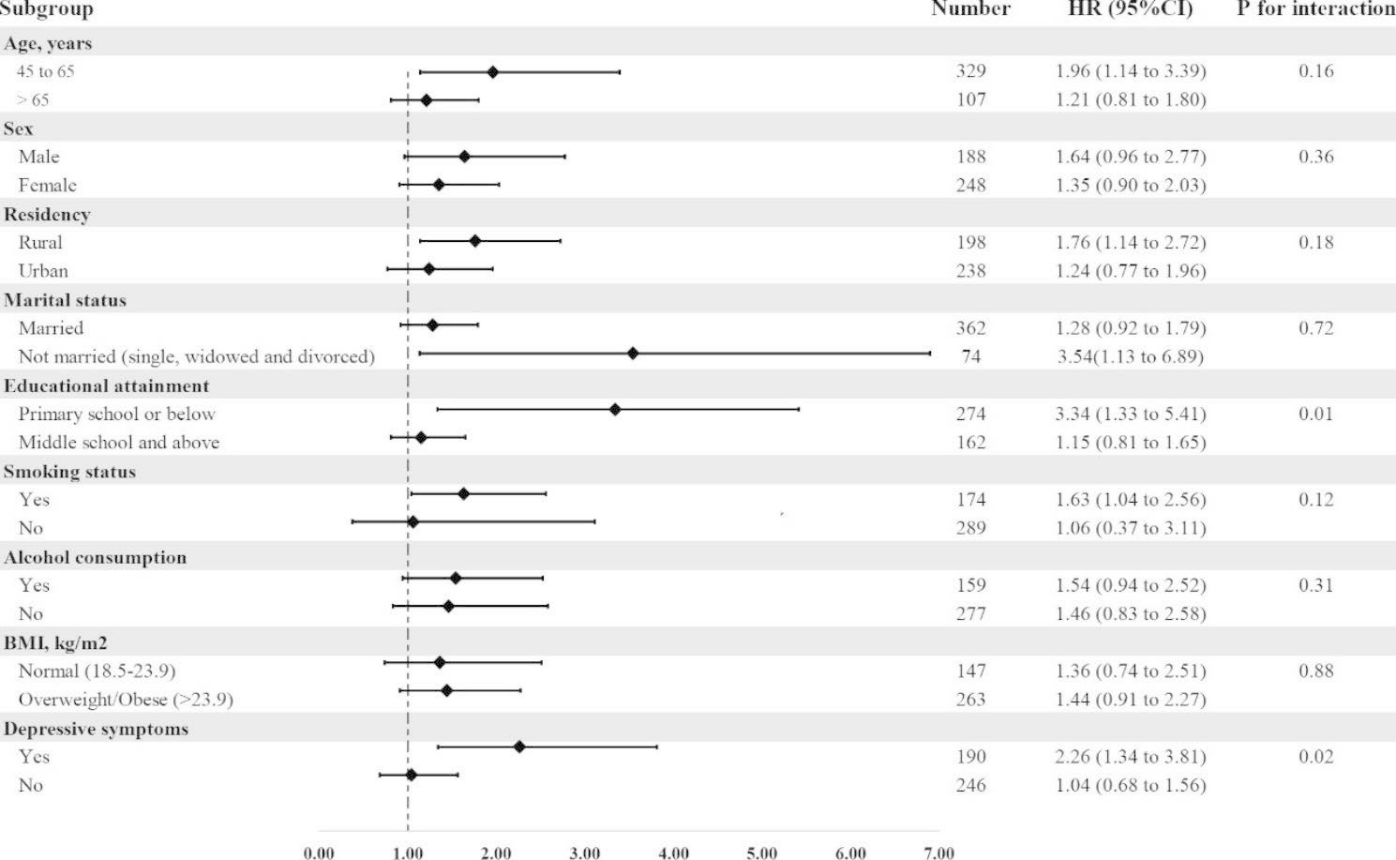
Subgroup analyses on the relationship between glycemic control and hypertension

We used a subgroup analysis to detect the effect of potential confounders, which may affect the relationship between glycemic control and incident hypertension. Figure 2 shows subgroup analyses on the relationship between diabetes with glycemic control and hypertension stratified by gender, residency, educational attainment, BMI, and depressive symptoms. The risk of hypertension differed in educational attainment subgroups (P for interaction = 0.01) and depressive symptoms subgroups (P for interaction = 0.02). Compared with diabetes with adequate glycemic control, those with insufficient glycemic control comorbid with depressive symptoms had a significantly higher risk of developing hypertension (HR = 2.26; 95% CI: 1.34 to 3.81). In educational attainment subgroups, the effect of glycemic control on hypertension occurred in diabetes with a diploma of primary school or below (HR = 3.34; 95% CI: 1.33 to 5.41).

## Discussion

This study examined the association between glycemic control and hypertension among middle-aged and older Chinese with diabetes. Several studies have demonstrated that higher blood glucose was an independent risk factor for hypertension. Derakhshan et al. found that prediabetic individuals were 1.25 times more likely to develop hypertension than euglycemic individuals [20]. Soon-Ki Ahn et al. also found similar results that those with diabetes had a 1.64 times higher risk of hypertension than nondiabetic one [10]. A previous systematic review showed that hypertension was common comorbidities in adults with diabetes [21]. Our findings extended the results of previous studies by showing that glycemic control was associated with a higher risk of hypertension among individuals with diabetes.

Several possible mechanisms may explain the relationship between glycemic control and hypertension. Function deficits of pancreatic β cells and insulin resistance could be indicated by the expression of HbA1c [22]. High levels of HbA1c were linked with proinflammatory cell signaling and oxidative stress, which may induce arterial stiffness [23]. In addition, increased levels of HbA1c can contribute to endothelial damage, promote the release of endothelin, and inhibit the production of nitric oxide and prostacyclin. These biochemical processes could lead to vasomotor dysfunction and increase blood pressure [24]. Clinical research found that blood lipids could be regulated by the high level of HbA1c, which leads to increased blood viscosity and a higher risk of cardiovascular diseases [24]. Moreover, epidemiological studies have found that hyperglycemia is frequently observed in frail hypertensive older adults [25, 26]. These findings indicated that reaching and maintaining optimal glycemic control may be crucial to reduce the incidence of hypertension and avoid complications.

We observed that the effect of glycemic control on hypertension was more pronounced among diabetes patients with lower educational attainment. Several studies have indicated a strong association between low education and cardiometabolic comorbidities and the evolution of chronic degenerative diseases [27–29]. The finding suggested that it is necessary to strengthen blood glucose monitoring among those with low education levels. The other meaningful result of this study was that diabetes with depressive symptoms and insufficient glycemic control had a significantly higher risk of developing hypertension. That is to say; mental health may influent the association between glycemic control and hypertension. The finding is in line with some other studies. A previous cohort study has revealed that anxiety and depressive symptoms predict the later incidence of hypertension and prescription treatment for hypertension [30]. A meta-analysis of prospective cohort studies showed that depression is probably an independent risk factor for hypertension. Diabetic patients with depressive symptoms can reduce their quality of life, minimize self-care ability, poorly control glycemic levels, and increase macrovascular and microvascular complications [31]. Therefore, our finding indicates that mental health should be part of diabetes management, and a psychiatrist or psychotherapist should be included in the diabetes management team.

This effect of glycemic control on the risk of hypertension is independent of age, gender, residency, education attainment, smoking, drinking, and BMI. In a cross-sectional study [32], a positive association between HbA1c and prevalent cardiovascular disease was observed. However, this was a univariate analysis and did not adjust for obesity. Notably, the associations of changes in weight or BMI and changes in blood pressure with hypertension were widely reported, and the significant association of the change in HbA1c level with incident hypertension was maintained even if controlled for BMI. The mechanism remains unclear, but our results suggest that the change in HbA1c might play a direct role in the increase in blood pressure through other mechanisms that are not entirely produced by weight gain [33, 34]. This highlights the importance of long-term monitoring of HbA1c levels.

Our study has several strengths. This is the first population-based cohort study to explore the association between glycemic control and hypertension among those with diabetes in China. Secondly, we use HbA1c to evaluate glycemic control rather than fasting plasma glucose (FPG). HbA1c has less biological variability and higher stability, and HbA1c could be less affected by relevant factors, such as acute infection, short-term lifestyle alterations, and recent eating behaviors. Moreover, FPG only reflects the immediate glycemia level at the time of a single measurement, whereas HbA1c is an indicator used to determine glycemic control in most diabetic patients across nearly two to 3 months [35]. Also, this study was subject to several limitations. First, CHARLS did not collect information on some confounders, such as family history, diary pattern, physical exercise, vascular damage, renal dysfunction, and information on other diseases. Although we examined the possible confounding effects of different variables on the association between the HbA1c level and the development of hypertension, there remained a possibility that unmeasured factors could have been confounders. Second, a possible limitation is selection bias due to missing data. Third, we used self-reported questionnaires to identify hypertension. Finally, we did not distinguish between type 1 and type 2 diabetes due to the limitation of data.

### Electronic supplementary material

Below is the link to the electronic supplementary material.

Supplementary Material 1

## Data Availability

The datasets generated and/or analyzed during the current study are available in the CHARLS repository, http://charls.pku.edu.cn/index/en.html (accessed on 3 September 2021).
